# Supplementary material for: Peptide Dendrimers with Non-Symmetric Bola Structure Exert Long Term Effect on Glioblastoma and Neuroblastoma Cell Lines
Source: Biomolecules. 2021 Mar 15;11(3):435. doi: 10.3390/biom11030435 (PMC8000084; doi:10.3390/biom11030435)
Supplement: Supplementary file 1 [file biomolecules-11-00435-s001.pdf]

Type of the Paper (Article)

Peptide dendrimers with non-symmetric bola structure exert long term effect on glioblastoma and neuroblastoma cell lines

Marta Sowińska[1#], MonikaSzeliga[2#],Maja Morawiak[1], Elżbieta Zieminska[2], Barbara Zabłocka[2] and Zofia Urbańczyk-Lipkowska[1,]

[1] Institute of Organic Chemistry PAS, 01-224 Warsaw, Poland; marta.sowinska@ryvu.com (M.S.) ; maja.morawiak@icho.edu.pl (M.M.); zofia.lipkowska@icho.edu.pl (Z.U.-L.);

[2] Mossakowski Medical Research Institute PAS, 02-106 Warsaw, Poland; mszeliga@imdik.pan.pl (M.Sz.); elziem@imdik.pan.pl (E.Z.); bzablocka@imdik.pan.pl (B.Z.)

\# equal contribution

* Correspondence: zofia.lipkowska@icho.edu.pl (Z.U.-L.);;

* Correspondence: mszeliga@imdik.pan.pl (M.Sz.);

Received: date; Accepted: date; Published: date

S1- Synthesis and analytical data

1. *General procedures*

All solvents and reagents were of analytical grade and were used without further purification. All solvents were obtained from Sigma-Aldrich (Steinheim, Germany). Mass spectra were recorded with a Mariner ESI time-of-flight mass spectrometer (PerSeptive Biosystems, Foster City, CA, USA) for the samples prepared in MeOH. The ^{1}H-NMR and ^{13}C-NMR spectra were recorded using a Bruker Avance spectrometer (Karlsruhe, Germany) at 500/125 or 400/100 MHz, respectively, using deuterated solvents and TMS as an internal standard. Chemical shifts are reported as δ values in parts per million, and coupling constants are given in hertz. The optical rotations (**$[\alpha]_D^{25}$**) were measured with JASCO J-1020 digital polarimeter (Ishikawa-machi, Hachioji, Tokyo, Japan). Melting points were recorded on a Köfler hot-stage apparatus (Wagner &Munz, München, Germany) and are uncorrected. Thin layer chromatography (TLC) was performed on aluminum sheets with silica gel 60 F254 from Merck ((Darmstadt, Germany). Column chromatography (CC) was carried out using silica gel (230–400 mesh) from Merck or Sephadex LH20 (Biosciences, Upsala, Sveden). The TLC spots were visualized by treatment with 1 % alcoholic solution of ninhydrin and heating.

2. *Synthesis and characterization of bola dendrimers*

The general strategy for the design of the studied series of the unsymmetrical bola-type dendrimers involved independent synthesis of the larger left side peptide dendrons functionalized at C-terminus with the appropriate linker, followed by coupling with smaller organic right side dendrons. The synthesis of Boc-protected right side organic dendrons functionalized with two p-aminobenzoic (PABA, **7**) or p-amino-benzenosulfonic acid (PAS, **8**) moieties, is shown in Scheme 1. Construction of the left side peptide dendron **10**, terminated with orthogonally protected lysines (e.g. 2-Cl-Z residue located at N^{α} and Boc groups at N^{ε} amino groups, respectively) from the intermediate **9** was performed according to the procedure outlined in Scheme 2. Further aminolysis of the C-terminal position in **10** with 2,2′-diethylamine (DEA) or 2,2′-(ethylenedioxy)diethylamine (NOON) yielded the

respective dendrons **11** and **12** (Scheme 2). Coupling with the linker required addition of high excess of the respective amines to a cooled to 0 °C methanolic solution of dendrons – 100 mols for EDA and 300 mols for NOON, respectively, and further titration for 5 days at r.t. or for 4 days at 60 °C, respectively for EDA and NOON. The resulting left side dendrons **11** and **12** equipped with the linkers were then coupled with the right side dendrons **7** and **8**. After Boc-deprotection of the amino groups with HCl/AcOEt solution, unsymmetrical bola-type molecules **16**, and **22** with shorter EDA linker and **18** and **24** with longer NOON linker were obtained in the form of hexa-hydrochlorides. Analogously, the synthesis of the left side intermediate **14** terminated with four Trp residues and extended with DEA linker from the previously described compound **13** is shown in Scheme 3. Example of the synthesis of the bola dendrimer **24** from intermediate **12** is shown in Scheme 4. The protocol for preparation of the bola structures: Boc-protected bola's **19** and **25** and the respective unprotected deca-hydrochlorides **20** and **26** are presented in Scheme 5 and 6, respectively. The unprotected bola dendrimers were obtained as creamy or pale yellow hygroscopic powders with no sharp melting point.

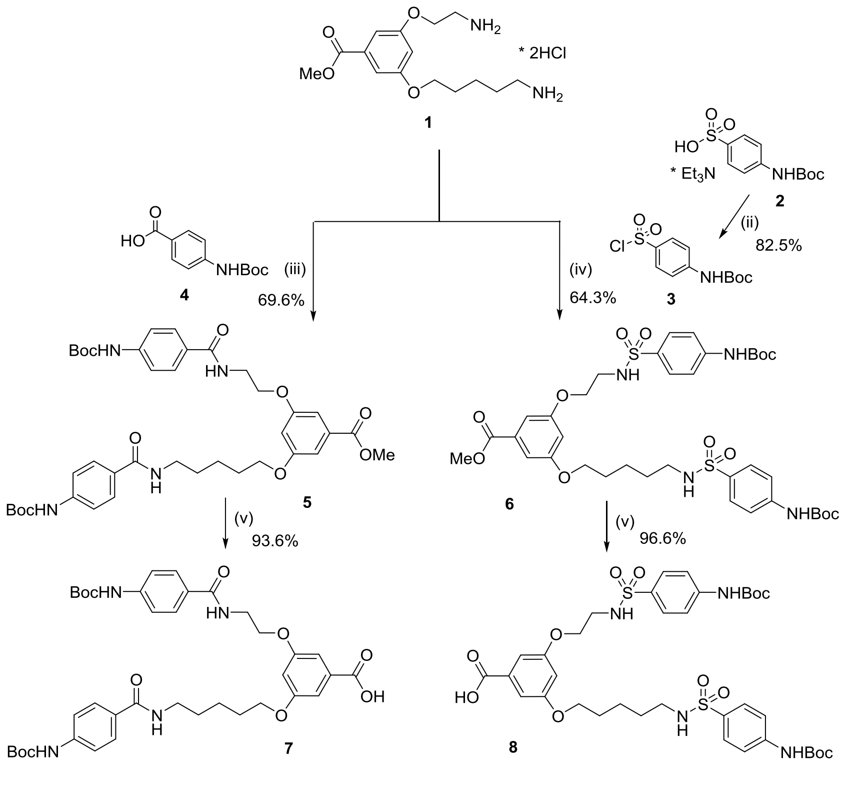

Scheme 1. Synthesis of the right side organic dendrons **7** and **8** with two PABA (**7**) or PAS (**8**) residues constituting right side of the bola structure starting from substrate **1** (synthesis of **1** described in ref 17). Conditions: .; (i) EDC/HOBt, Et_3N, DMF, 96h; (ii) EDC/HOBt, Et_3N, CH_2Cl_2/DMF, r.t..; 48 h; (iii) 1) 1M NaOH, MeOH, 50 °C; 2) 1M HCl.

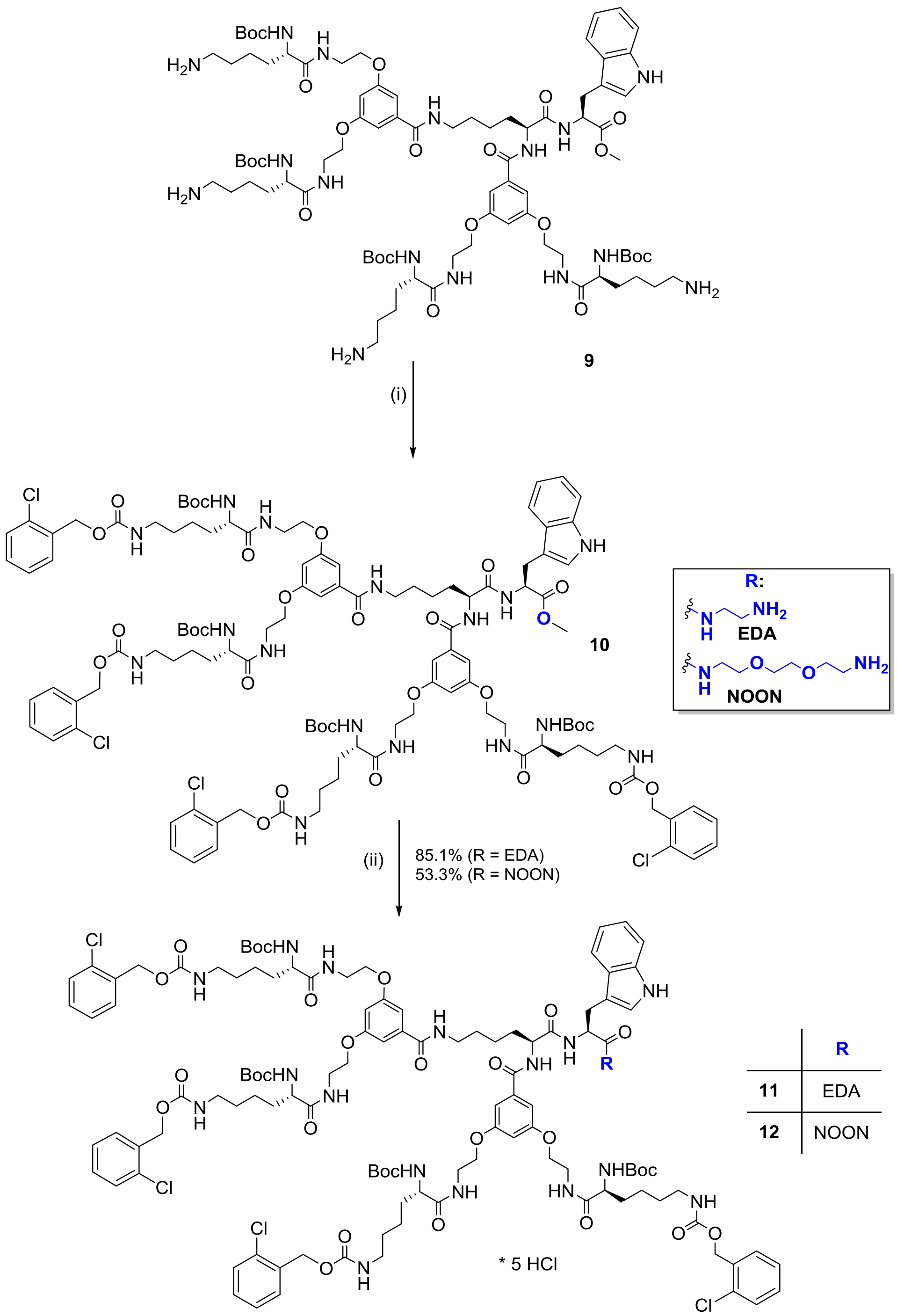

Scheme 2. Synthesis of 2-Cl-Z terminated dendrimers **12** and **13** constituting linker-terminated peptidic left side of the bola structure starting from substrate **9** (synthesis of substrate **9** described in ref. 13). Conditions: (i) Boc-Lys(2-Cl-Z)-OH, DCC/HOBt, Et3N, DMF, 72h (ii) EDA, MeOH, r.t., 10 days, or NOON, MeOH, 60 °C, 4 days.

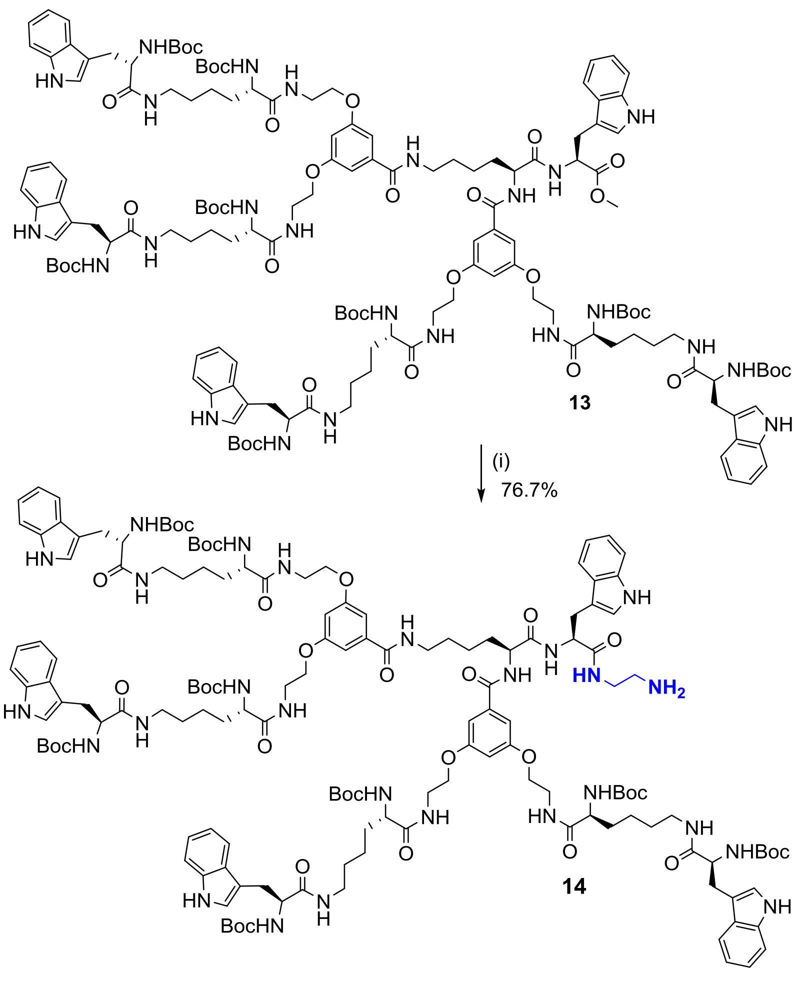

Scheme 3. Synthesis of Trp terminated dendron **14** with EDA spacer constituting left side of the bola structure (**14** described in ref. 13).

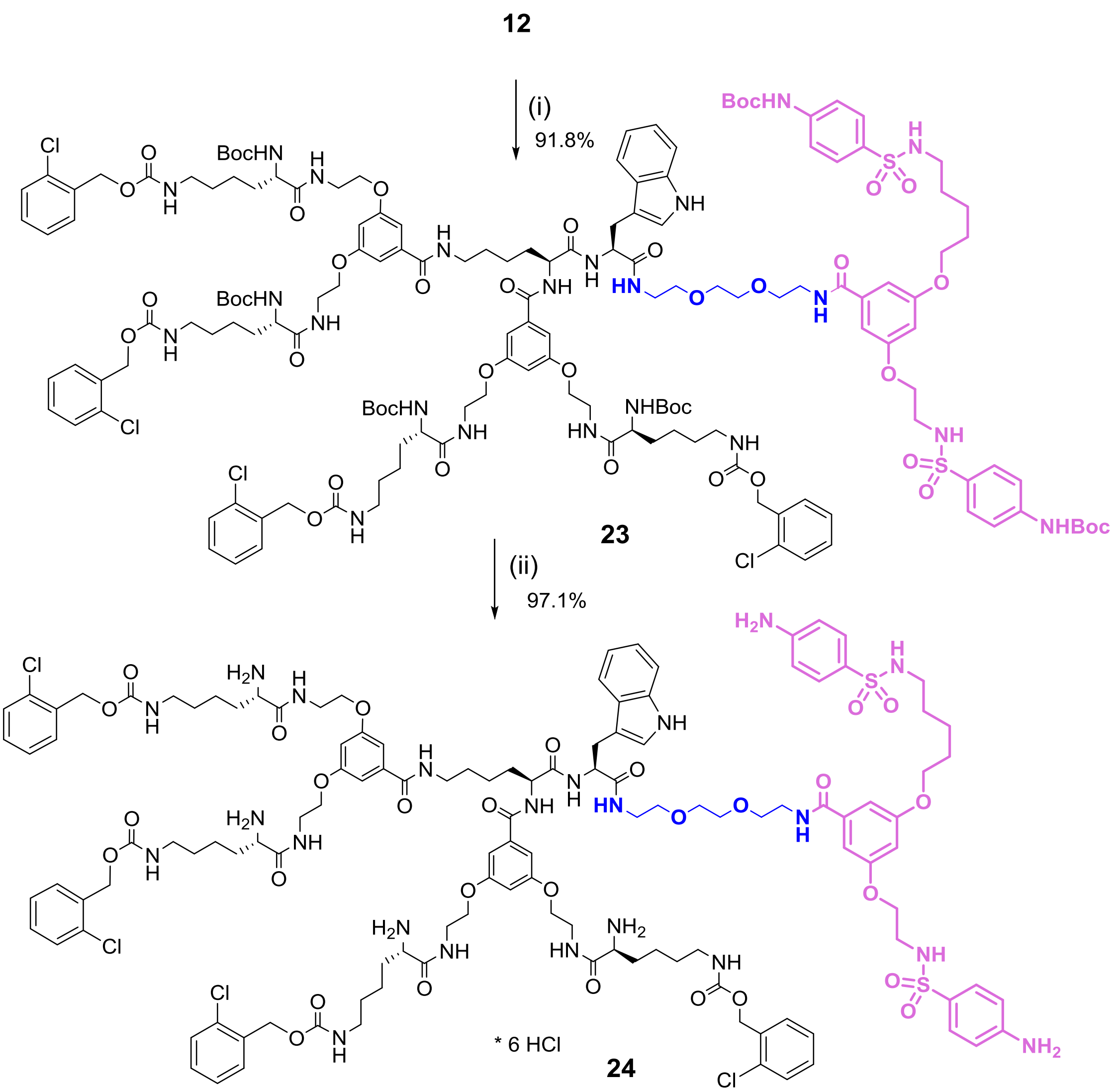

Scheme 4. Synthesis of bola dendrimer **24** from intermediate **12** with left side Dendron terminated with 2-chloro-benzyloxycarbonyl (2-Cl-Z) residues and right side Dendron functionalized with *p*-aminosulfonic acid (PAS). Conditions: (i) (i) Boc-Lys(2-Cl-Z)-OH, DCC/HOBt, Et3N, DMF, 72h (ii) sat HCl/EtOAc.

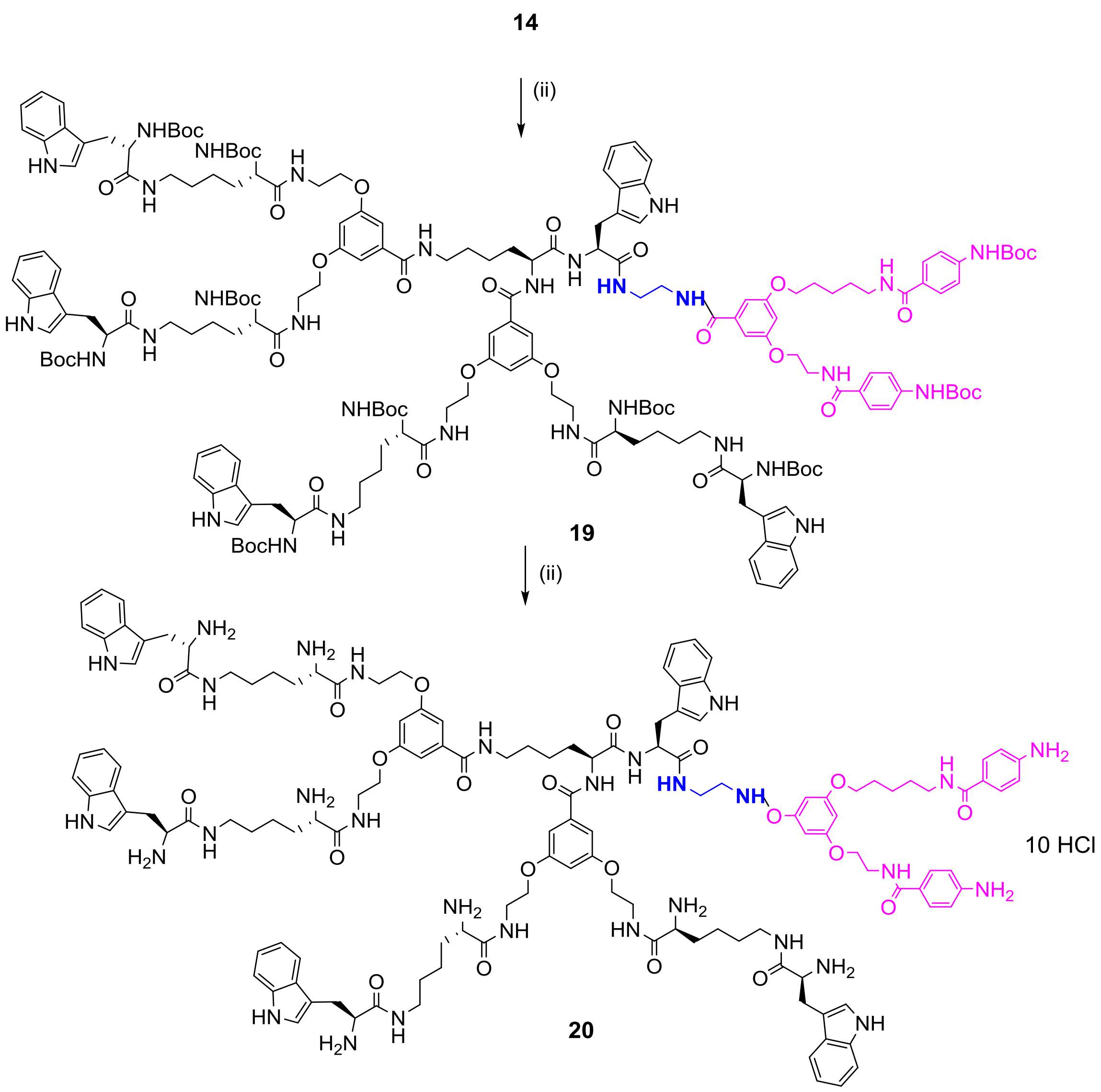

Scheme 5. Synthesis of Trp-terminated dendrimer **20** containing EDA linker/spacer. (i) **7**, EDC/HOBt, DMF, 1.9 eq. Et_3N 10 days; (ii) sat. HCl/EtOAc.

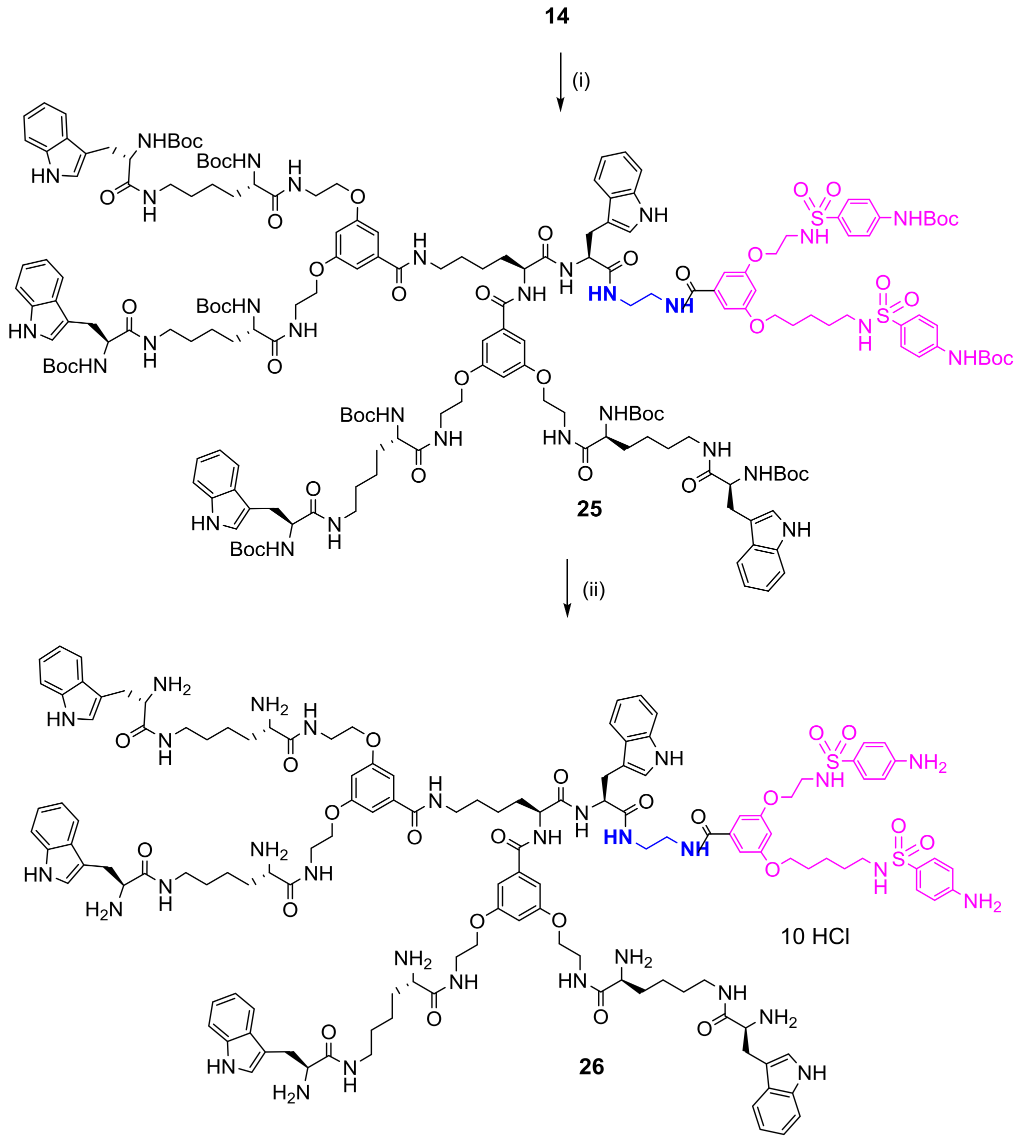

Scheme 6. Synthesis of Boc-protected compound **25** and unprotected compound **26**. Conditions: (i) EDC/HOBt, Et_3N, DMF, 5 days; (ii) sat. HCl/EtOAc

Table 1. Structure of the obtained dendrons and bola dendrimers.

Dendron 1 (left side) + dendron 2 (rightside) —(i)→ bola - dendrimer (protected) —(ii)→ bola-dendrimer (unprotected)

(i) EDC/HOBt, Et_3N, DMF, 3 – 5 days; (ii) sat. HCl/EtOAc.

**Dendron 1**<br>**Side groups+linker**	**„Dendron 2”**	**Dendrimer bola**<br>**(Boc-protected)**	**Dendrimer bola**<br>**(deprotected)**<br>**M.W. (g/mol)**
(2-Cl-Z)<br>**11**<br>EDA/	7<br>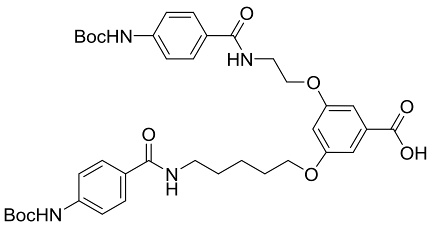 	11+7=**15**	**16**<br>2727.28
(2-Cl-Z) NOON<br>**12**		12+7=**17**	**18**<br>2815.39
(Trp) EDA<br>**14**		**14+7=19**	**20**<br>2943.66
(2-Cl-Z) **119** EDA<br>**11**	8<br>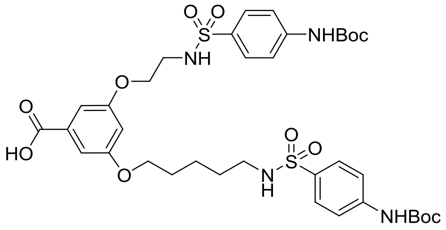 	11+8=**21**	**22**<br>2799.39
(2-Cl-Z) NOON<br>**12**		12+8=**23**	**24**<br>2887.50
(Trp) EDA<br>**14**		**14+8=25**	**26**<br>3015.77

3. Spectrocscopic data of new dendrons 7, 8 and bola dendrimers 16 – 26

3.1 Dendrons

Dendron 7

White powder, yield 93.6% (0.67 g).

$C_{38}H_{48}O_{10}N_4$, M = 720.81 g/mol (monoisotopic mass 720.3); **R_f** = 0.27 ($CHCl_3$/MeOH 8:1). **M.p.:** 199-200˚C.

Dendron 8

Creamy powder, yield 96.6% (0.85 g).

$C_{36}H_{48}O_{12}N_4S_2$, M = 792.92 g/mol (monoisotopic mass 792.3). **LRMS** (ESI, MeOH):815.5 [M + Na^+]. **R_f** = 0.35 ($CHCl_3$/MeOH 8:1).**M.p.:** 99-101˚C.

3.2. Dendrimers

3.2.1. N-Protected bola dendrimers

General coupling procedure with EDC/HOBt

To a solution of acid in DMF (1.1 equiv. per one amino group of dendrimer) HOBt was added (1.1 equiv. per one amino group of dendrimer), then cooled to 0 ^{o}C and stirred under argon atmosphere at for 5 min. Then, suspension of EDC·HCl (1.1 equiv. per one amino group of dendrimer) and Et_3N in quantity required for neutralization of hydrochloride were added and stirred for 50 min. To this mixture 1 eq. of dendrimer was added. The reaction was continued in r.t. and under normal atmosphere. Reaction was moniterred using TLC and Kaiser test. A solution was filtered and evaporated to dryness. The residue was dissolved in DCM and washed with 2% citric acid solution, saturated aqueous $NaHCO_3$ and brine, dried over $MgSO_4$, filtered and evaporated *in vacuo*. After DMF evaporation raw dendrimeric compound was purified by molecular filtration on the Sephadex LH-20 packing in MeOH. and then by silica gel chromatography. The above general procedure was applied to obtain six N-protected bola dendrimers. Better yields were achieved when an additional amount of Et_3N (0.9 equiv.), was added to a reaction mixture to improve transformation of coupling reagent (EDC·HCl) to its free form (usually 1,1 equiv.) Reaction was accomplished in 3-5 days. After DMF evaporation, reaction mixture was dissolved in MeOH and size exclusion chromatography with the use of Sephadex LH-20 and MeOH as eluent was applied. Next column chromatography using silica gel $CHCl_3$/MeOH (25:1 for dendrimers **15**, **16**; 15:1 for **21**, **22**; and gradient 100:1→10:1 for **19**, **25**) were applied yielding dendrimeric compounds as creamy or pale yellow powders.

a) Dendrimer 15

Creamy powder, 85.9% (0.85 g).

$C_{155}H_{204}O_{37}N_{22}Cl_4$, M = 3109.22 g/mol (monoisotopic mass 3105.4).

LRMS (ESI, MeOH):3132.1 [M + Na$^+$], 1576.1 [M + 2Na$^+$]$^{2+}$ - *main signal.*

^{1}**H NMR** (500 MHz, MeOD):δ = 1.20-1.39 [br m, 10H, 4×γCH_2*L-Lys*, O-$(CH_2)_2$-C***H₂***-$(CH_2)_2$-NH] overlapped with 1.37 [s, 36H, 4×$C(CH_3)_3$*L-Lys*], 1.41-1.51 [br m, 12H, 4×δCH_2*L-Lys*, γCH_2*core*, O-$(CH_2)_3$-C***H₂***-CH_2-NH] overlapped with 1.49, 1.50 [2s, 18H, 2×$C(CH_3)_3$ *PABA*], 1.53-1.76 [br m, 14H, 5×βCH_2 *L-Lys and core*, δCH_2*core*, O-CH_2-C***H₂***-$(CH_2)_3$-NH], 3.03 (m, 8H, 4×εCH_2*L-Lys*), 3.14 (m, 1H, βCH_2*L-Trp*), 3.25 [m, 5H, βCH_2*L-Trp*, εCH_2*core*, O-$(CH_2)_4$-C***H₂***-NH], 3.34 (m, 4H, NH-C***H₂***-C***H₂***-NH), 3.42-3.60 (br m, 8H, 4×O-CH_2-C***H₂***-NH), 3.68 (m, 2H, O-CH_2-C***H₂***-NH), 3.84-4.12 (br m, 16H, 6×O-CH_2, 4×αCH

L-Lys), 4.35 (m, 1H, αCH *core*), 4.58 (m, 1H, αCH *L-Trp*), 5.11 (2s, 8H, Ar-C$\boldsymbol{H_2}$O), 6.59 (m, 3H, C^4-H *Ph*), 6.87-7.04 (br m, 9H, $C^{2,6}$-H *Ph*, $C^{2,5,6}$-H *L-Trp*), 7.23 (m, 9H, ArH, C^7-H *L-Trp*), 7.29-7.40 (2m, 8H, ArH), 7.46 (m, 5H, C^4-H *L-Trp*, $C^{3,5}$-H *PABA*), 7.71 (m, 4H, $C^{2,6}$-H *PABA*).

^{13}C NMR (500 MHz, MeOD): δ = 24.1 [γC, O-$(CH_2)_2$-***C***H_2-$(CH_2)_2$-NH], 28.3 (βC *L-Trp*), 28.7, 28.8 [C(***C***H_3)$_3$], 30.0, 30.5 [δC,O-CH_2-***C***H_2-CH_2-***C***H_2-CH_2-NH], 33.2 (βC), 39.9, 40.6 (6×CH_2NH, NH-***C***H_2-***C***H_2-NH), 40.7 (εC *core*), 41.5 (4×εC *L-Lys*), 54.9 (αC *L-Trp*), 56.1 (4×αC *L-Lys*), 56.3 (αC *core*), 64.6 (Ar-***C***H_2O), 67.7 (5×O-***C***H_2-CH_2-NH), 69.2 [O-***C***H_2-$(CH_2)_4$-NH], 80.7, 81.2 [***C***$(CH_3)_3$], 105.9, 106.0 (C^4*Ph*), 106.9, 107.3, 107.5 ($C^{2,6}$*Ph*), 112.4 (C^7*L-Trp*), 118.8 ($C^{3,5}$*PABA*), 119.3 (C^4*L-Trp*), 119.9 (C^5*L-Trp*), 122.6 (C^6*L-Trp*), 124.5 (C^2*L-Trp*), 128.1 (CH_{Ar}), 129.2 ($C^{2,6}$*PABA*), 129.3 (C^{3a}*L-Trp*), 130.4 (CH_{Ar}), 134.1 (C_{Ar}-Cl), 135.9 (***C***$_{Ar}$-CH_2O), 144.0 (C^4*PABA*), 158.56 [C=O (Boc, 2-Cl-Z)], 161.3 ($C^{3,5}$*Ph*), 175.5 (CONH).

$[\alpha]_D^{25}$= -13.7 (c 1, MeOH).

R_f = 0.37 ($CHCl_3$/MeOH 8:1).

M.p.: 119-126°C.

b) Dendrimer 17

Pale yellow powder yield 82.8% (0.53 g).

$C_{159}H_{212}O_{39}N_{22}Cl_4$, M = 3197.32 g/mol (monoisotopic mass 3193.4).

LRMS (ESI, MeOH):3220.3 [M + Na^+], 1620.0 [M + $2Na^+$]$^{2+}$ - *main signal*.

^{1}H NMR (500 MHz, MeOD):δ = 1.27-1.77 [br m, 36H, 5×β, γ, δCH_2 *L-Lys and core*,O-CH_2-(C$\boldsymbol{H_2}$)$_3$-CH_2-NH] overlapped with 1.37, 1.49 [3s,54H, 6×C$(CH_3)_3$], 3.04 (m, 8H, 4×εCH_2*L-Lys*),3.10-3.40 [br m, 10H, βCH_2*L-Trp*, εCH_2*core*, O-$(CH_2)_4$-C$\boldsymbol{H_2}$-NH, 2×C$\boldsymbol{H_2}$NH *NOON*], 3.43-3.61 (br m, 16H, 4×O-CH_2-C$\boldsymbol{H_2}$-NH,4×OCH_2*NOON*),3.71 (m, 2H, O-CH_2-C$\boldsymbol{H_2}$-NH), 3.89 [m, 2H, O-C$\boldsymbol{H_2}$-$(CH_2)_4$-NH], 4.00 (br m, 12H, 4×αCH *L-Lys*, 4×O-C$\boldsymbol{H_2}$-CH_2-NH), 4.11 (m, 2H, O-C$\boldsymbol{H_2}$-CH_2-NH), 4.45 (m, 1H, αCH *core*), 4.62 (m, 1H, αCH *L-Trp*), 5.11 (2s, 8H, Ar-C$\boldsymbol{H_2}$O), 6.60 (m, 3H, C^4-H *Ph*), 6.89-7.02 (br m, 8H, $C^{2,6}$-H *Ph*, $C^{5,6}$-H *L-Trp*), 7.05 (s, 1H, C^2-H *L-Trp*), 7.24 (m, 9H, ArH, C^7-H *L-Trp*), 7.35 (2m, 8H, ArH), 7.47 (d, *J* = 8.62Hz, 4H, $C^{3,5}$-H *PABA*), 7.51 (d, *J* =7.94 Hz, 1H, C^4-H *L-Trp*), 7.73 (m, 4H, $C^{2,6}$-H *PABA*).

^{13}C NMR (500 MHz, MeOD):δ = 24.1, 24.4, 24.6 [γC, O-$(CH_2)_2$-***C***H_2-$(CH_2)_2$-NH], 28.7, 28.8 [C(***C***H_3)$_3$], 29.0 (βC *L-Trp*), 29.9, 30.1, 30.3, 30.5, 30.7[δC,O-CH_2-***C***H_2-CH_2-***C***H_2-CH_2-NH], 32.4, 33.0, 33.2 (βC), 39.9 (4×O-CH_2-***C***H_2-NH), 40.4, 40.6, 40.7, 40.9, 41.0 [εC *core*, O-$(CH_2)_4$-***C***H_2-NH, O-CH_2-***C***H_2-NH, 2×CH_2NH *NOON*], 41.5 (4×εC *L-Lys*), 55.8(αC *core*, αC

L-Trp), 56.1 (4×αC *L-Lys*), 64.6 (Ar-***C***H_2O), 67.7, 67.8 (5×O-***C***H_2-CH_2-NH), 69.1 [O-***C***H_2-$(CH_2)_4$-NH], 70.3, 70.5, 71.2, 71.3 (4×OCH_2*NOON*), 80.6, 81.3 [***C***$(CH_3)_3$], 105.7, 105.8, 106.1 (C^4*Ph*), 107.0, 107.2, 107.3, 107.5 ($C^{2,6}$*Ph*), 110.8 (C^3*L-Trp*), 112.4 (C^7*L-Trp*), 118.8 ($C^{3,5}$*PABA*), 119.5 (C^4*L-Trp*), 119.9 (C^5*L-Trp*), 122.5 (C^6*L-Trp*), 124.7 (C^2*L-Trp*), 128.1 (CH_{Ar}), 128.8 (C^{3a}*L-Trp*), 128.9 (C^1*PABA*), 129.2, 129.3 ($C^{2,6}$*PABA*), 130.4 (CH_{Ar}), 134.1 (C_{Ar}-Cl), 135.9 (***C***$_{Ar}$-CH_2O), 137.1, 137.6(C^1*Ph*), 137.9 (C^{7a}*L-Trp*),143.9, 144.1 (C^4*PABA*), 154.8, 157.8, 158.5 [C=O (Boc, 2-Cl-Z)], 161.2, 161.4, 161.7 ($C^{3,5}$*Ph*), 169.6, 169.7, 169.8, 169.9(CONH *Ph*), 173.6, 174.1, 175.5 (CONH).

$[\alpha]_D^{25}$= -10.5 (c 1, MeOH).

R_f = 0.30 ($CHCl_3$/MeOH 8:1).

M.p.: 110-119°C.

c) Dendrimer 19

Creamy powder, yield 89.2% (0.91 g).

$C_{187}H_{256}O_{41}N_{30}$, M = 3580.21 g/mol (monoisotopic mass 3577.9).

LRMS (ESI, MeOH):1812.5 $[M + 2Na^+]^{2+}$, 1816.9 $[M + MeOH + H^+ + Na^+]^{2+}$ - *main signal.*

^{1}H NMR (500 MHz, MeOD):δ = 1.0-1.77 [br m, 36H,5×β, γ, δCH_2 *L-Lys and core,* O-CH_2-(C***H***$_2$)$_3$-CH_2-NH] overlapped with 1.33, 1.37, 1.49, 1.50 [4s, 90H, 10×$C(CH_3)_3$], 2.8-3.4 [br m, 26H, 5×εCH_2*L-Lys and core*, 5×βCH_2*L-Trp*, O-$(CH_2)_4$-C***H***$_2$-NH, NH-C***H***$_2$-C***H***$_2$-NH], 3.47, 3.56 (2m, 8H, 4×O-CH_2-C***H***$_2$-NH), 3.68 (m, 2H, O-CH_2-C***H***$_2$-NH), 3.85 [m, 2H, O-C***H***$_2$-$(CH_2)_4$-NH], 3.95 (m, 12H, 4×O-C***H***$_2$-CH_2-NH, 4×αCH *L-Lys*), 4.08 (m, 2H, O-C***H***$_2$-CH_2-NH), 4.28 (m, 4H, 4×αCH*L-Trp*), 4.37 (m, 1H, αCH *core*), 4.58 (m, 1H, αCH*L-Trp*), 6.59 (m, 3H, C^4-H *Ph*), 6.88-7.07 (br m, 21H, $C^{2,6}$-H *Ph*, $C^{2,5,6}$-H *L-Trp*),7.22 (d, *J* = 8.03 Hz, 1H, C^7-H *L-Trp*), 7.29 (m, 4H, C^7-H *L-Trp*), 7.47(m, 5H, C^4-H *L-Trp*, $C^{3,5}$-H *PABA*), 7.54(m, 4H, C^4-H *L-Trp*), 7.72 (m, 4H, $C^{2,6}$-H *PABA*).

^{13}C NMR (500 MHz, MeOD):δ= 24.2, 24.4, 24.6 [γC, O-$(CH_2)_2$-***C***H_2-$(CH_2)_2$-NH], 28.4(βC *L-Trp*), 28.7, 28.8 [C(***C***H_3)$_3$], 29.6, 29.8, 29.9 (4×βC *L-Trp*), 30.1, 30.3, 30.7 [δC, 2×O-CH_2-***C***H_2-CH_2-***C***H_2-CH_2-NH], 32.1, 33.1, 33.2 (βC), 40.0, 40.1, 40.4, 40.6, 40.7, 40.9 (εC, 6×CH_2NH, NH-C***H***$_2$-C***H***$_2$-NH), 56.0 (αC *L-Trp*, αC *core*), 56.1 (4×αC *L-Lys*), 57.1 (4×αC *L-Trp*), 67.7 (5×O-***C***H_2-CH_2-NH), 69.1 [O-***C***H_2-$(CH_2)_4$-NH],80.6, 81.3 [***C***$(CH_3)_3$], 105.8, 105.9, 106.2 (C^4*Ph*), 106.8, 107.2, 107.4 ($C^{2,6}$*Ph*), 110.9, 111.1 (C^3*L-Trp*), 112.3, 112.4 (C^7*L-Trp*), 118.8 ($C^{3,5}$*PABA*), 119.3, 119.5 (C^4*L-Trp*), 119.8, 119.9 (C^5*L-Trp*), 122.4, 122.6 (C^6*L-Trp*), 124.6 (C^2*L-Trp*), 128.7, 128.8 (C^{3a}*L-Trp*), 128.9 (C^1*PABA*), 129.2, 129.4 ($C^{2,6}$*PABA*), 137.5, 137.9(C^1*Ph*), 138.0 (C^{7a}*L-Trp*),143.9, 144.1 (C^4*PABA*), 154.8, 157.5, 157.8 [C=O (Boc)],

161.2, 161.3, 161.7 ($C^{3,5}$*Ph*), 169.7, 169.9(CONH *Ph*), 170.1, 174.3, 174.5, 174.6, 175.5 (CONH).

$[\alpha]_D^{25}$= -7.0 (c 1, MeOH).

R_f = 0.43 ($CHCl_3$/MeOH 8:1).

M.p.: 146-151˚C.

d) Dendrimer 21

Creamy powder, yield 85.5% (0.71 g).

$C_{153}H_{204}O_{39}N_{22}Cl_4S_2$, M = 3181.32 g/mol (monoisotopic mass 3177.3).

LRMS (ESI, MeOH):3205.2 [M + Na^+], 1611.6 $[M + 2Na^+]^{2+}$ - *main signal*.

^{1}H NMR (500 MHz, MeOD):δ = 1.20-1.51 [br m, 22H, 5×γCH_2*L-Lys and core*, 4×δCH_2*L-Lys*, O-$(CH_2)_2$-C***H₂***-C***H₂***-CH_2-NH] overlapped with 1.37 [s, 36H, 4×$C(CH_3)_3$*L-Lys*] i 1.49 [s, 18H, 2×$C(CH_3)_3$*SA*], 1.58 [br m, 8H, 2×βCH_2, δCH_2*core*, O-CH_2-C***H₂***-$(CH_2)_3$-NH], 1.64-1.83 (brm, 6H, 3×βCH_2), 2.79 (br t, 2H, O-$(CH_2)_4$-C***H₂***-NH], 3.03 (m, 8H, 4×εCH_2*L-Lys*), 3.17 (m, 3H,βCH_2*L-Trp*, O-CH_2-C***H₂***-$NHSO_2$), 3.25 (m, 3H,βCH_2*L-Trp*, εCH_2*core*), 3.34 (m, 4H, NH-C***H₂***-C***H₂***-NH), 3.42-3.62 (br m, 8H, 4×O-CH_2-C***H₂***-NH), 3.80 [m, 2H, O-C***H₂***-$(CH_2)_4$-NH], 3.88-4.07 (brm, 14H, 5×O-C***H₂***-CH_2-NH, 4×αCH *L-Lys*), 4.36 (m, 1H, αCH *core*), 4.59 (m, 1H, αCH *L-Trp*), 5.12 (2s, 8H, Ar-C***H₂***O), 6.42 (m, 1H, C^4-H *Ph*), 6.62 (m, 2H, C^4-H *Ph*), 6.84-7.01 (br m, 8H, $C^{2,6}$-H *Ph*, $C^{5,6}$-H *L-Trp*), 7.04 (s, 1H, C^2-H *L-Trp*), 7.24 (m, 9H, ArH,C^7-H *L-Trp*), 7.30-7.41(2m, 8H, ArH), 7.48-7.60 (br m, 5H, C^4-H *L-Trp*, $C^{3,5}$-H *SA*), 7.70 (m, 4H, $C^{2,6}$-H *SA*).

^{13}C NMR (500 MHz, MeOD): δ = 24.1, 24.2[γC, O-$(CH_2)_2$-***C***H_2-$(CH_2)_2$-NH], 28.3 (βC *L-Trp*), 28.7, 28.8 [C(***C***H_3)$_3$], 29.8, 30.1, 30.3, 30.5[δC,O-CH_2-***C***H_2-CH_2-***C***H_2-CH_2-NH], 33.2 (βC), 39.9 (4×O-CH_2-***C***H_2-NH), 40.4, 40.7. 40.8 (εC *core*, NH-***C***H_2-***C***H_2-NH), 41.5(4×εC *L-Lys*), 43.5 (O-CH_2-***C***H_2-$NHSO_2$), 44.0[O-$(CH_2)_4$-***C***H_2-NH],56.0 (αC *L-Trp*), 56.1 (4×αC *L-Lys*), 56.3 (αC *core*), 64.6 (Ar-***C***H_2O), 67.8 (4×O-***C***H_2-CH_2-NH), 68.1(O-***C***H_2-CH_2-$NHSO_2$), 69.1 [O-***C***H_2-$(CH_2)_4$-NH], 80.7, 81.5 [***C***$(CH_3)_3$], 105.9, 106.4, 106.7 (C^4*Ph*), 107.3, 107.5 ($C^{2,6}$*Ph*), 110.9 (C^3*L-Trp*), 112.5 (C^7*L-Trp*), 119.0 ($C^{3,5}$*SA*), 119.3 (C^4*L-Trp*), 119.9 (C^5*L-Trp*), 122.6 (C^6*L-Trp*), 124.6 (C^2*L-Trp*), 128.1 (CH_{Ar}), 128.7 (C^{3a}*L-Trp*), 129.1 ($C^{2,6}$*SA*), 130.4, 130.5 (CH_{Ar}), 134.1 (C_{Ar}-Cl), 134.7 (C^1*SA*), 135.9 (***C***$_{Ar}$-CH_2O), 137.4, 137.9(C^1*Ph*), 138.0 (C^{7a}*L-Trp*), 144.9 (C^4*SA*), 154.6, 157.8, 158.5 [C=O (Boc, 2-Cl-Z)], 161.2, 161.3, 161.5 ($C^{3,5}$*Ph*), 169.9(CONH *Ph*), 174.3, 174.4, 175.5 (CONH).

$[\alpha]_D^{25}$= -11.6 (c 1, MeOH).

R_f = 0.30 ($CHCl_3$/MeOH 8:1).

M.p.: 114-122˚C.

e) Dendrimer 23

Pale yellow powder, yield 91.8% (0.78 g).

$C_{157}H_{212}O_{41}N_{22}Cl_4S_2$, M = 3269.43 g/mol (monoisotopic mass 3265.3).

LRMS (ESI, MeOH):3288.6[M + Na^+], 1655.2 [M + 2Na^+]$^{2+}$ - *main signal.*

^{1}H NMR (500 MHz, MeOD):δ = 1.28-1.75 [br m, 36H, 5×β, γ, δCH_2 *L-Lys and core*,O-CH_2-(C***H₂***)$_3$-CH_2-NH] overlapped with 1.37, 1.39 [3s, 54H, 6×C(CH_3)$_3$], 2.81 [m, 2H, O-(CH_2)$_4$-C$\boldsymbol{H_2}$-NH], 3.04 (m, 8H, 4×εCH_2 *L-Lys*),3.10-3.35 (br m, 10H, βCH_2*L-Trp*, εCH_2*core*, O-CH_2-C$\boldsymbol{H_2}$-NHSO_2, 2×C$\boldsymbol{H_2}$NH *NOON*), 3.40-3.62 (br m, 16H, 4×O-CH_2-C$\boldsymbol{H_2}$-NH,4×OCH_2*NOON*), 3.82 [m, 2H, O-C$\boldsymbol{H_2}$-(CH_2)$_4$-NH], 3.92 (m, 2H, O-C$\boldsymbol{H_2}$-CH_2-NHSO_2), 4.00 (br m, 12H, 4×O-C$\boldsymbol{H_2}$-CH_2-NH, 4×αCH *L-Lys*), 4.48 (m, 1H, αCH *core*), 4.61 (m, 1H, αCH *L-Trp*), 5.12 (2s, 8H, Ar-C$\boldsymbol{H_2}$O), 6.42 (m, 1H, C^4-H *Ph*), 6.62 (m, 2H, C^4-H *Ph*), 6.82-6.98 (br m, 8H, $C^{2,6}$-H *Ph*, $C^{5,6}$-H *L-Trp*), 7.06 (s, 1H, C^2-H *L-Trp*), 7.24 (m, 9H, ArH, C^7-H *L-Trp*), 7.36 (2m, 8H, ArH), 7.55 (m, 5H, C^4-H *L-Trp*, $C^{3,5}$-H *SA*), 7.71 (m, 4H, $C^{2,6}$-H *SA*).

^{13}C NMR (500 MHz, MeOD): δ = 24.1, 24.2 [γC, O-(CH_2)$_2$-$\boldsymbol{C}H_2$-(CH_2)$_2$-NH], 28.7, 28.8 [C($\boldsymbol{C}H_3$)$_3$], 29.0 (βC *L-Trp*), 29.8, 30.1, 30.3, 30.5 [δC,O-CH_2-$\boldsymbol{C}H_2$-CH_2-$\boldsymbol{C}H_2$-CH_2-NH], 32.4, 33.2 (βC), 40.0 (4×O-CH_2-$\boldsymbol{C}H_2$-NH), 40.5, 40.7, 41.0 (εC *core*, 2×CH_2NH *NOON*), 41.5 (4×εC *L-Lys*), 43.5 (O-CH_2-$\boldsymbol{C}H_2$-NHSO_2), 44.0 [O-(CH_2)$_4$-$\boldsymbol{C}H_2$-NH], 55.8 (αC *L-Trp*, αC *core*), 56.1 (4×αC *L-Lys*), 64.6 (Ar-$\boldsymbol{C}H_2$O), 67.8 (4×O-$\boldsymbol{C}H_2$-CH_2-NH), 68.0 (O-$\boldsymbol{C}H_2$-CH_2-NHSO_2), 69.1 [O-$\boldsymbol{C}H_2$-(CH_2)$_4$-NH], 70.3, 70.5, 71.3 (4×OCH_2*NOON*), 80.6, 81.5 [$\boldsymbol{C}$(CH_3)$_3$], 105.6, 105.9, 106.2 (C^4*Ph*), 106.7, 107.2, 107.5 ($C^{2,6}$*Ph*), 110.8 (C^3*L-Trp*), 112.4 (C^7*L-Trp*), 119.0 ($C^{3,5}$*SA*), 119.5 (C^4*L-Trp*), 119.9 (C^5*L-Trp*), 122.5 (C^6*L-Trp*), 124.8 (C^2*L-Trp*), 128.2 (CH_{Ar}), 128.8 (C^{3a}*L-Trp*), 129.1 ($C^{2,6}$*SA*), 130.4, 130.5 (CH_{Ar}), 134.1 (C_{Ar}-Cl), 134.7, 134.8 (C^1*SA*), 135.9 ($\boldsymbol{C}_{Ar}$-CH_2O), 137.5 (C^1*Ph*), 137.9 (C^{7a}*L-Trp*), 144.9 (C^4*SA*), 154.6, 157.8, 158.5 [C=O (Boc, 2-Cl-Z)], 161.0, 161.2, 161.3, 161.6 ($C^{3,5}$*Ph*), 169.8 (CONH *Ph*), 173.7, 174.1, 175.5 (CONH).

$[\alpha]_D^{25}$= -9.6 (c 1, MeOH).

R_f = 0.52 ($CHCl_3$/MeOH 8:1).

M.p.: 109-115°C.

f) Dendrimer 25

Creamy powder, yield 85.7% (0.54 g).

$C_{185}H_{256}O_{43}N_{30}S_2$, M = 3652.32 g/mol (monoisotopic mass 3649.8).

LRMS (ESI, MeOH):3677.1 [M + Na^+], 1848.0 [M + 2Na^+]$^{2+}$ - *main signal*, 1239.6 [M + 3Na^+]$^{3+}$.

^{1}H NMR (500 MHz, MeOD):δ = 1.0-1.81 [br m, 36H, 5×β, γ, δCH_2 *L-Lys and core*, O-CH_2-(C***H$_2$***)$_3$-CH_2-NH] overlapped with 1.34, 1.38, 1.49 [3s, 90H, 10×$C(CH_3)_3$], 2.78 [m, 2H, O-$(CH_2)_4$-C***H$_2$***-NH], 2.8-3.4 (br m, 26H, 5×εCH_2*L-Lys and core*, 5×βCH_2*L-Trp,* NH-C***H$_2$***-C***H$_2$***-NH, O-CH_2-C***H$_2$***-$NHSO_2$), 3.42-3.62 (2m, 8H, 4×O-CH_2-C***H$_2$***-NH), 3.75-3.90 [br m, 4H, O-C***H$_2$***-$(CH_2)_4$-NH, O-C***H$_2$***-CH_2-$NHSO_2$], 3.97 (m, 12H, 4×αCH *L-Lys*, 4×O-C***H$_2$***-CH_2-NH), 4.29 (m, 4H, 4×αCH *L-Trp*), 4.37 (m, 1H, αCH *core*), 4.59 (t, 1H, αCH *L-Trp*), 6.42 (m, 1H, C^4-H *Ph*), 6.61 (m, 2H, C^4-H *Ph*), 6.80-7.08 (br m, 21H, $C^{2,6}$-H *Ph*, $C^{2,5,6}$-H *L-Trp*), 7.24 (d, *J* = 8.1 Hz, 1H, C^7-H *L-Trp*), 7.30 (d, *J* = 7.7 Hz, 4H, C^7-H *L-Trp*), 7.47-7.60 (br m, 9H, C^4-H *L-Trp*, $C^{3,5}$-H *SA*), 7.70 (m, 4H, $C^{2,6}$-H *SA*).

^{13}C NMR (500 MHz, MeOD): δ = 24.2 [γC, 2×O-$(CH_2)_2$-***C***H_2-$(CH_2)_2$-NH], 28.4(βC *L-Trp*), 28.7, 28.8 [C(***C***H_3)$_3$], 29.6, 29.7, 29.8 (4×βC *L-Trp*), 30.1, 30.2, 30.3, 30.5, 30.7 [δC, 2×O-CH_2-***C***H_2-CH_2-***C***H_2-CH_2-NH], 33.1, 33.2 (βC), 40.0 (4×O-CH_2-***C***H_2-NH), 40.1, 40.4, 40.7 (εC *core*, NH-***C***H_2-***C***H_2-NH), 42.4 (4×εC *L-Lys*), 43.5 (O-CH_2-***C***H_2-$NHSO_2$), 44.0 [O-$(CH_2)_4$-***C***H_2-NH], 56.0 (αC *L-Trp*), 56.1 (4×αC *L-Lys*), 56.3 (αC *core*), 57.1 (4×αC *L-Trp*), 67.8 (4×O-***C***H_2-CH_2-NH), 68.0 (O-***C***H_2-CH_2-$NHSO_2$), 69.1 [O-***C***H_2-$(CH_2)_4$-NH], 79.5, 80.6, 81.5 [***C***$(CH_3)_3$], 105.9, 106.3 (C^4*Ph*), 106.6, 107.2, 107.4 ($C^{2,6}$*Ph*), 110.9, 111.1 (C^3*L-Trp*), 112.3, 112.4 (C^7*L-Trp*), 119.0 ($C^{3,5}$*SA*), 119.3, 119.5 (C^4*L-Trp*), 119.8, 119.9 (C^5*L-Trp*), 122.5, 122.6 (C^6*L-Trp*), 124.6 (C^2*L-Trp*), 128.7, 128.8 (C^{3a}*L-Trp*), 129.1 ($C^{2,6}$*SA*), 134.6, 134.7 (C^1*SA*), 137.4, 137.9 (C^1*Ph*), 138.0 (C^{7a}*L-Trp*), 144.9 (C^4*SA*), 154.6, 157.5, 157.8 [C=O (Boc)],161.0, 161.2, 161.3, 161.5 ($C^{3,5}$*Ph*), 169.9 (CONH *Ph*), 170.1, 174.4, 174.6, 175.5 (CONH).

$[\alpha]_D^{25}$= -6.7 (c 1, MeOH).

R_f = 0.53 ($CHCl_3$/MeOH 8:1).

M.p.: 123-130°C.

3.2.2. Unprotected dendrimers

Boc-deprotection of dendrimers **15**, **17**, **19**, and **21**, **23**, **25** was performed with saturated HCl/AcOEt solution. Typically, 0.3 – 0.4 g (0.08 - 0.122 mmol) of dendrimer was dissolved in 1 or 2 mL MeOH and mixed with 15 mL of saturated HCl/AcOEt for 2 h. Complete Boc-deprotection yielded either hexahydrochlorides (**16**, **18**, **22**, **24**) or decahydrochlorides (**20**, **26**) as hygroscopic pale yellow powders

a) Dendrimer 16

Dendrimer **16** was obtained from 0.3 g (0.096 mmol) of **15** as pale yellow powder; yield 92.3% (0.24 g).

$C_{125}H_{156}O_{25}N_{22}Cl_4$×6HCl, M = 2727.28 g/mol (monoisotopic mass of non-protonated product – 2505.0).

LRMS (ESI, MeOH):1280.5 [M + MeOH + H^+ + Na^+]$^{2+}$, 1253.6 [M + $2H^+$]$^{2+}$, 1169.6 [M – 2ClZ + $2H^+$]$^{2+}$, 853.7 [M + MeOH + $2H^+$ + Na^+]$^{3+}$, 836.1 [M + $3H^+$]$^{3+}$ - *main signal*, 798.0 [M – 2ClZ + MeOH + $2H^+$ + Na^+]$^{3+}$, 780.0 [M – 2ClZ + $3H^+$]$^{3+}$.

^{1}H NMR (500 MHz, MeOD):δ = 1.30-1.90 [br m, 36H, 5×β, γ, δCH_2 *L-Lys and core*,O-CH_2-(C***H$_2$***)$_3$-CH_2-NH], 2.85 (m, 2H, εCH_2core), 3.04 (m, 8H, 4×εCH_2 *L-Lys*), 3.12-3.4 [br m, 8H, βCH_2*L-Trp*, O-$(CH_2)_4$-C***H$_2$***-NH, NH-C***H$_2$***-C***H$_2$***-NH], 3.53 (m, 4H, 2×O-CH_2-C***H$_2$***-NH), 3.70 (m, 6H, 3×O-CH_2-C***H$_2$***-NH), 3.91 [m, 6H, O-C***H$_2$***-$(CH_2)_4$-NH, 4×αCH *L-Lys*], 4.08 (m, 8H, 4×O-C***H$_2$***-CH_2-NH), 4.16 (m, 2H, O-C***H$_2$***-CH_2-NH), 4.35 (m, 1H, αCH *core*), 4.59 (m, 1H, αCH *L-Trp*), 5.11 (2s, 8H, Ar-C***H$_2$***O), 6.60-6.71 (br m, 3H, C^4-H *Ph*), 6.90-7.05 (br m, 8H, $C^{2,6}$-H *Ph*, $C^{5,6}$-H *L-Trp*), 7.13 (m, 1H, C^2-H *L-Trp*), 7.25 (m, 9H, ArH, C^7-H *L-Trp*), 7.36 (m, 8H, ArH), 7.47 (m, 5H, C^4-H *L-Trp*, $C^{3,5}$-H *PABA*), 7.94 (d, *J* = 8.14 Hz, 4H, $C^{2,6}$-H *PABA*).

^{13}C NMR (500 MHz, MeOD): δ = 23.0, 24.5 [γC, 2×O-$(CH_2)_2$-***C***H_2-$(CH_2)_2$-NH], 28.1 (βC *L-Trp*), 29.9, 30.1, 30.5 [δC,2×O-CH_2-***C***H_2-CH_2-***C***H_2-CH_2-NH], 32.1, 32.3 (βC), 40.2, 40.3, 40.7, 41.0 (εC *core*, 6×CH_2-NH, NH-C***H$_2$***-C***H$_2$***-NH), 41.3 (4×εC *L-Lys*), 54.2, 54.5 (4×αC *L-Lys*), 56.0 (αC *L-Trp*), 56.5 (αC *core*), 64.7 (Ar-***C***H_2O), 67.7 (5×O-***C***H_2-CH_2-NH), 69.2 [O-***C***H_2-$(CH_2)_4$-NH], 105.9, 106.0 (C^4*Ph*), 106.5, 106.8, 107.3, 107.6 ($C^{2,6}$*Ph*), 112.4 (C^7*L-Trp*), 119.3 (C^4*L-Trp*), 119.9 (C^5*L-Trp*), 122.5 (C^6*L-Trp*), 124.1, 124.2 ($C^{3,5}$*PABA*), 124.6 (C^2*L-Trp*), 128.2 (CH_{Ar}), 128.7 (C^{3a}*L-Trp*), 130.3 ($C^{2,6}$*PABA*), 130.4, 130.5 (CH_{Ar}), 132.3 (C^1*PABA*), 134.1 (C_{Ar}-Cl), 135.8 (***C***$_{Ar}$-CH_2O), 136.4 (C^4*PABA*), 137.5 (C^1*Ph*), 138.0 (C^{7a}*L-Trp*), 158.6 [C=O (2-Cl-Z)], 161.2, 161.3, 161.7 ($C^{3,5}$*Ph*), 168.9 (CONH *Ph*), 170.3, 170.4 (CONH).

$[\alpha]_D^{25}$= -10.0 (c 1, MeOH).

M.p.: 162-175°C.

b) Dendrimer 18

Dendrimer **18** was obtained from 0.31 g (0.097 mmol) of **17**as creamy powder; yield 96.3% (0.26 g).

$C_{129}H_{164}O_{27}N_{22}Cl_4$×6HCl, M = 2815.39 g/mol (monoisotopic mass of non-protonated product – 2593.1).

LRMS (ESI, MeOH):2594.2 [M + H^+], 1297.4 [M + $2H^+$]$^{2+}$, 1324.9 [M + MeOH + H^+ + Na^+]$^{2+}$, 651.3 [M + $4H^+$]$^{4+}$ - *main signal*.

^{1}H NMR (500 MHz, MeOD):δ = 1.30-1.92 [br m, 36H, 5×β, γ, δCH_2 *L-Lys and core*,O-CH_2-(C***H$_2$***)$_3$-CH_2-NH], 2.85 (m, 2H, εCH_2*core*), 3.03 (m, 8H, 4×εCH_2*L-Lys*), 3.10-3.60[br m, 20H, βCH_2*L-Trp*, O-$(CH_2)_4$-C***H$_2$***-NH, 2×O-CH_2-C***H$_2$***-NH, 2×C***H$_2$***NH *NOON*, 4×OCH_2*NOON*), 3.70 (br m, 6H, 3×O-CH_2-C***H$_2$***-NH), 3.91 [m, 6H, O-C***H$_2$***-$(CH_2)_4$-NH, 4×αCH *L-Lys*], 4.08

(m, 8H, 4×O-C***H₂***-CH₂-NH), 4.16 (m, 2H, O-C***H₂***-CH₂-NH), 4.34 (m, 1H, αCH *core*), 4.59 (m, 1H, αCH *L-Trp*), 5.11 (2s, 8H, Ar-C***H₂***O), 6.60-6.71 (br m, 3H, C^4-H *Ph*), 6.89-7.04 (br m, 8H, $C^{2,6}$-H *Ph*, $C^{5,6}$-H *L-Trp*), 7.07 (s, 1H, C^2-H *L-Trp*), 7.25 (m, 9H, ArH, C^7-H *L-Trp*), 7.36 (m, 8H, ArH), 7.47 (m, 5H, C^4-H *L-Trp*, $C^{3,5}$-H *PABA*), 7.94 (d, *J* = 8.14 Hz, 4H, $C^{2,6}$-H *PABA*).

^{13}C NMR (500 MHz, MeOD): δ = 23.0, 24.5 [γC, 2×O-$(CH_2)_2$-***C***H_2-$(CH_2)_2$-NH], 29.0 (βC *L-Trp*), 29.9, 30.1, 30.2, 30.5 [δC,2×O-CH_2-***C***H_2-CH_2-***C***H_2-CH_2-NH], 32.3, 32.5 (βC), 40.2 (4×O-CH_2-***C***H_2-NH), 40.4, 40.6, 40.8, 40.9 [εC *core*, O-$(CH_2)_4$-***C***H_2-NH, O-CH_2-***C***H_2-NH, 2×CH_2NH *NOON*], 41.3 (4×εC *L-Lys*), 54.2, 54.4 (4×αC *L-Lys*), 55.8 (αC *L-Trp*), 56.0 (αC *core*), 64.7 (Ar-***C***H_2O), 67.6, 67.8 (5×O-***C***H_2-CH_2-NH), 69.1 [O-***C***H_2-$(CH_2)_4$-NH], 70.3, 70.5, 71.2 (4×OCH_2*NOON*), 105.6, 105.8, 106.2 (C^4*Ph*), 106.9, 107.3, 107.5 ($C^{2,6}$*Ph*), 110.8 (C^3*L-Trp*), 112.4 (C^7*L-Trp*), 119.2 (C^4*L-Trp*), 119.9 (C^5*L-Trp*), 122.5 (C^6*L-Trp*), 124.1, 124.2 ($C^{3,5}$*PABA*), 124.8 (C^2*L-Trp*), 128.2 (CH_{Ar}), 128.8 (C^{3a}*L-Trp*), 130.1, 130.2 ($C^{2,6}$*PABA*), 130.4, 130.5 (CH_{Ar}), 134.1 (C_{Ar}-Cl), 135.8 (***C***$_{Ar}$-CH_2O), 137.2, 137.5 (C^1*Ph*), 137.9 (C^{7a}*L-Trp*), 158.6 [C=O (2-Cl-Z)], 161.2, 161.4, 161.7 ($C^{3,5}$*Ph*), 169.4, 169.5, 169.8, 169.9 (CONH *Ph*), 170.4, 173.7, 174.1 (CONH).

$[\alpha]_D^{25}$= -5.1 (c 1, MeOH).

M.p.: 149-155°C.

c) Dendrimer 20

Dendrimer **20** was obtained from 0.3 g (0.08 mmol) of **19** as pale yellow powder; yield 97.2% (0.24 g).

$C_{137}H_{176}O_{21}N_{30}$×10HCl, M = 2943.66 g/mol (monoisotopic mass of non-protonated product – 2577.4).

LRMS (ESI, MeOH):1289.7 $[M + 2H^+]^{2+}$, 860.2 $[M + 3H^+]^{3+}$ - *main signal*, 645.4 $[M + 4H^+]^{4+}$.

^{1}H NMR (500 MHz, MeOD):δ = 1.25-1.81 [br m, 36H, 5×β, γ, δCH_2 *L-Lys and core*,O-CH_2-(C***H₂***)$_3$-CH_2-NH], 2.92-3.40 [br m, 26H, 5×εCH_2 *L-Lys and core*, 5×βCH_2*L-Trp*, O-$(CH_2)_4$-C***H₂***-NH, NH-C***H₂***-C***H₂***-NH], 3.54 (m, 4H, 2×O-CH_2-C***H₂***-NH), 3.60-3.74 (br m, 6H, 3×O-CH_2-C***H₂***-NH), 3.89 [m, 6H, O-C***H₂***-$(CH_2)_4$-NH, 4×αCH *L-Lys*], 4.01-4.16 (br m, 14H, 5×O-C***H₂***-CH_2-NH, 4×αCH *L-Trp*), 4.37 (m, 1H, αCH *core*), 4.58 (m, 1H, αCH *L-Trp*), 6.59-6.69 (m, 3H, C^4-H *Ph*), 6.88-7.12 (br m, 17H, $C^{2,6}$-H *Ph*, $C^{2,5,6}$-H *L-Trp*), 7.20 (s, 4H, C^2-H *L-Trp*), 7.25 (d, *J* = 8.09 Hz, 1H, C^7-H *L-Trp*), 7.36 (d, *J* = 8.07 Hz, 4H, C^7-H *L-Trp*), 7.47 (m, 5H, C^4-H *L-Trp*, $C^{3,5}$-H *PABA*), 7.64 (m, 4H, C^4-H *L-Trp*), 7.94 (m, 4H, $C^{2,6}$-H *PABA*).

^{13}C NMR (500 MHz, MeOD): δ = 23.2, 24.5 [γC, 2×O-$(CH_2)_2$-***C***H_2-$(CH_2)_2$-NH], 28.9(βC *L-Trp*), 29.6, 29.9, 30.1 [δC,2×O-CH_2-***C***H_2-CH_2-***C***H_2-CH_2-NH], 32.3 (βC), 40.1, 40.2, 40.7, 41.0 (εC, 6×CH_2-NH, NH-C***H_2***-C***H_2***-NH), 54.4 (4×αC *L-Lys*), 55.2 (4×αC *L-Trp*), 56.0 (αC *L-Trp*), 56.1 (αC *core*), 67.7 (5×O-***C***H_2-CH_2-NH), 69.2 [O-***C***H_2-$(CH_2)_4$-NH], 105.8, 106.0 (C^4*Ph*), 106.3, 106.7, 107.3, 107.6 ($C^{2,6}$*Ph*), 108.1, 110.9 (C^3*L-Trp*), 112.4, 112.6 (C^7*L-Trp*), 119.4 (C^4*L-Trp*), 119.9, 120.2 (C^5*L-Trp*), 122.5, 122.9 (C^6*L-Trp*), 124.2, 124.3 ($C^{3,5}$*PABA*), 125.7 (C^2*L-Trp*), 128.4 (C^{3a}*L-Trp*), 130.3, 130.4 ($C^{2,6}$*PABA*), 135.9 (C^1*PABA*), 136.4, 136.9 (C^4*PABA*),137.4, 137.9 (C^1*Ph*), 138.2 (C^{7a}*L-Trp*), 161.2, 161.3, 161.8 ($C^{3,5}$*Ph*), 168.6, 168.9, 169.6 (CONH *Ph*), 170.0, 170.1, 170.5 (CONH).

$[\alpha]_D^{25}$= +11.2 (c 1, MeOH).

M.p.: 209-220°C.

d) Dendrimer 22

Dendrimer **22** was obtained from 0.3 g (0.094 mmol) of **21** as creamy powder; yield 96.2% (0.25 g).

$C_{123}H_{156}O_{27}N_{22}Cl_4S_2$×6HCl, M = 2799.39 g/mol (monoisotopic mass of non-protonated product – 2577.0).

LRMS (ESI, MeOH):1316.5[M + MeOH + Na^+ + $H^+]^{2+}$, 1289.6 [M + $2H^+]^{2+}$, 1205.6 [M – 2ClZ + $2H^+]^{2+}$, 878.3 [M + MeOH + Na^+ + $2H^+]^{3+}$, 860.0 [M + $3H^+]^{3+}$ - *main signal*, 804.0 [M – 2ClZ + $3H^+]^{3+}$.

^{1}H NMR (500 MHz, MeOD):δ = 1.34-1.72 [br m, 26H, 5×γ, δCH_2*L-Lys and core*, O-CH_2-(C***H_2***)$_3$-CH_2-NH], 1.84 (br m, 10H, 5×βCH_2*L-Lys and core*), 2.84 [m, 4H, O-$(CH_2)_4$-C***H_2***-NH, εCH_2*core*], 3.04 (m, 8H, 4×εCH_2*L-Lys*), 3.15-3.45 (br m, 8H, βCH_2*L-Trp*, O-CH_2-C***H_2***-$NHSO_2$, NH-C***H_2***-C***H_2***-NH), 3.53, 3.68 (2m, 8H, 4×O-CH_2-C***H_2***-NH), 3.90 [br m, 8H, O-C***H_2***-$(CH_2)_4$-NH, O-C***H_2***-CH_2-$NHSO_2$, 4×αCH *L-Lys*], 4.08 (m, 8H, 4×O-C***H_2***-CH_2-NH), 4.33 (m, 1H, αCH *core*), 4.59 (m, 1H, αCH *L-Trp*), 5.11 (2s, 8H, Ar-C***H_2***O), 6.51 (m, 1H, C^4-H *Ph*), 6.63-6.78 (br m, 3H, C^4-H *Ph*, $C^{2,6}$-H *Ph*), 6.93 (m, 2H, $C^{2,6}$-H *Ph*, C^5-H *L-Trp*), 7.01 (m, 5H, $C^{2,6}$-H *Ph*, C^6-H *L-Trp*), 7.10 (s, 1H, C^2-H *L-Trp*), 7.26 (m, 9H, ArH, C^7-H *L-Trp*), 7.30-7.55 (br m, 13H, ArH, C^4-H *L-Trp*, $C^{3,5}$-H *SA*), 7.91 (m, 4H, $C^{2,6}$-H *SA*).

^{13}C NMR (500 MHz, MeOD): δ = 23.0, 24.2, 24.4 [γC, 2×O-$(CH_2)_2$-***C***H_2-$(CH_2)_2$-NH], 28.1 (βC *L-Trp*), 29.7, 30.1, 30.3, 30.4 [δC,2×O-CH_2-***C***H_2-CH_2-***C***H_2-CH_2-NH], 32.0, 32.1, 32.3 (βC), 40.2, 40.3, 40.8 (εC *core*, 4×O-CH_2-***C***H_2-NH, NH-C***H_2***-C***H_2***-NH), 41.3 (4×εC *L-Lys*), 43.5 (O-CH_2-***C***H_2-$NHSO_2$), 44.0 [O-$(CH_2)_4$-***C***H_2-NH], 54.3, 54.5 (4×αC *L-Lys*), 56.0 (αC *L-Trp*), 56.6 (αC *core*), 64.7 (Ar-***C***H_2O), 67.7 (4×O-***C***H_2-CH_2-NH), 68.1 (O-***C***H_2-CH_2-$NHSO_2$), 69.2 [O-***C***H_2-$(CH_2)_4$-NH], 105.8, 106.0, 106.5 (C^4*Ph*), 107.1, 107.3, 107.6 ($C^{2,6}$*Ph*), 110.9

(C^3*L-Trp*), 112.5 (C^7*L-Trp*), 119.3 (C^4*L-Trp*), 119.9 (C^5*L-Trp*), 122.5 (C^6*L-Trp*), 123.5, 123.6 ($C^{3,5}$*SA*), 124.7 (C^2*L-Trp*), 128.2 (CH_{Ar}), 128.7 (C^{3a}*L-Trp*), 130.0 ($C^{2,6}$*SA*), 130.4, 130.5 (CH_{Ar}), 134.1 (C_{Ar}-Cl), 135.8 (***C***$_{Ar}$-CH_2O), 136.9 (C^1*SA*), 137.4 (C^1*Ph*), 138.0 (C^{7a}*L-Trp*), 140.5 (C^4*SA*), 158.6 [C=O (2-Cl-Z)], 161.0, 161.2, 161.3, 161.6 ($C^{3,5}$*Ph*), 170.0, 170.4, 174.4 (CONH).

$[\alpha]_D^{25}$= -9.6 (c 1, MeOH).

M.p.: 148-162˚C.

e) Dendrimer 24

Dendrimer **24** was obtained from 0.4 g (0.122 mmol) of **23** as pale yellow powder; yield 97.1% (0.34 g).

$C_{127}H_{164}O_{29}N_{22}Cl_4S_2$×6HCl, M = 2887.50 g/mol (monoisotopic mass of non-protonated product – 2665.0).

LRMS (ESI, MeOH):1344.6 $[M + H^+ +Na^+]^{2+}$, 1333.6 $[M + 2H^+]^{2+}$, 1249.6 $[M – (2\text{-Cl-Z}) + 2H^+]^{2+}$, 889.4 $[M + 3H^+]^{3+}$ - *main signal,* 833.4 $[M – (2\text{-Cl-Z}) + 3H^+]^{3+}$.

^{1}H NMR (500 MHz, MeOD):δ = 1.30-1.90 [br m, 36H, 5×β, γ, δCH_2 *L-Lys and core*, O-CH_2-(C***H₂***)$_3$-CH_2-NH], 2.85 [m, 4H, O-$(CH_2)_4$-C***H₂***-NH, εCH_2*core*], 3.03 (m, 8H, 4×εCH_2*L-Lys*),3.10-3.36 (br m, 8H, βCH_2*L-Trp*, O-CH_2-C***H₂***-NHSO_2, 2×C***H₂***NH *NOON*), 3.40-3.74 (br m, 16H, 4×O-CH_2-C***H₂***-NH, 4×OCH_2*NOON*), 3.88 [m, 6H, O-C***H₂***-$(CH_2)_4$-NH, 4×αCH *L-Lys*], 3.94 (m, 2H, O-C***H₂***-CH_2-NHSO_2), 4.08 (br m, 8H, 4×O-C***H₂***-CH_2-NH), 4.46 (m, 1H, αCH *core*), 4.61 (m, 1H, αCH *L-Trp*), 5.12 (2s, 8H, Ar-C***H₂***O), 6.52 (m, 1H, C^4-H *Ph*), 6.67 (m, 2H, C^4-H *Ph*), 6.75 (m, 1H, $C^{2,6}$-H *Ph*), 6.92 (m, 2H, $C^{2,6}$-H *Ph*, C^5-H *L-Trp*), 7.02 (m, 5H, $C^{2,6}$-H *Ph*, C^6-H *L-Trp*), 7.10 (s, 1H, C^2-H *L-Trp*), 7.26 (m, 9H, ArH, C^7-H *L-Trp*), 7.33-7.44 (br m, 12H, ArH, $C^{3,5}$-H *SA*), 7.52 (d, *J* = 7.93 Hz, 1H, C^4-H *L-Trp*), 7.90 (m, 4H, $C^{2,6}$-H *SA*).

^{13}C NMR (500 MHz, MeOD): δ = 23.0, 24.2, 24.6 [γC, 2×O-$(CH_2)_2$-***C***H_2-$(CH_2)_2$-NH], 29.0 (βC *L-Trp*), 29.7, 30.1, 30.4, 30.5 [δC,2×O-CH_2-***C***H_2-CH_2-***C***H_2-CH_2-NH], 32.3 (βC), 40.2 (4×O-CH_2-***C***H_2-NH), 40.4, 40.8, 41.0 (εC *core*, 2×CH_2NH *NOON*), 41.3 (4×εC *L-Lys*), 43.5 (O-CH_2-***C***H_2-NHSO_2), 44.0 [O-$(CH_2)_4$-***C***H_2-NH], 54.4 (4×αC *L-Lys*), 55.8 (αC *L-Trp*), 56.0 (αC *core*), 64.7 (Ar-***C***H_2O), 67.6 (4×O-***C***H_2-CH_2-NH), 68.1 (O-***C***H_2-CH_2-NHSO_2), 69.1 [O-***C***H_2-$(CH_2)_4$-NH], 70.3, 70.5, 71.2 (4×OCH_2*NOON*), 105.5, 105.8, 106.3 (C^4*Ph*), 107.0, 107.1, 107.3, 107.5 ($C^{2,6}$*Ph*), 110.7 (C^3*L-Trp*), 112.4 (C^7*L-Trp*), 119.4 (C^4*L-Trp*), 119.9 (C^5*L-Trp*), 122.5 (C^6*L-Trp*), 122.9, 123.1 ($C^{3,5}$*SA*), 124.8 (C^2*L-Trp*),128.2 (CH_{Ar}), 128.8 (C^{3a}*L-Trp*), 130.0 ($C^{2,6}$*SA*), 130.4, 130.5 (CH_{Ar}), 134.1 (C_{Ar}-Cl), 135.8 (***C***$_{Ar}$-CH_2O), 137.2, 137.5

(C^1*Ph*), 137.9 (C^{7a}*L-Trp*), 158.6 [C=O (2-Cl-Z)], 161.0, 161.2, 161.3, 161.7 ($C^{3,5}$*Ph*), 169.5 (CONH *Ph*), 170.0, 170.4, 173.7, 174.1 (CONH).

$[\alpha]_D^{25}$= -6.3 (c 1, MeOH).

M.p.: 139-147˚C.

f) Dendrimer 26

Dendrimer **26** was obtained from 0.34 g (0.09 mmol) of **25** as pale yellow powder; yield 89.3% (0.25 g).

$C_{135}H_{176}O_{23}N_{30}S_2$×10HCl, M = 3015.77 g/mol (monoisotopic mass of non-protonated product – 2649.3).

LRMS (ESI, MeOH):1352.7 $[M + MeOH + H^+ + Na^+]^{2+}$, 1325.7 $[M + 2H^+]^{2+}$, 902.1 $[M + MeOH + 2H^+ + Na^+]^{3+}$, 884.1 $[M + 3H^+]^{3+}$ - *main signal*, 690.3 $[M + 2MeOH + 2H^+ + 2Na^+]^{4+}$, 676.8 $[M + MeOH + 3H^+ + Na^+]^{4+}$, 663.3 $[M + 4H^+]^{4+}$.

^{1}H NMR (500 MHz, MeOD):δ = 1.25-1.65 [br m, 26H, 5×γ, δCH_2*L-Lys and core,* O-CH_2-($C\boldsymbol{H_2}$)$_3$-CH_2-NH], 1.78 (m, 10H, 5×βCH_2*L-Lys and core*), 2.81 [m, 4H, O-$(CH_2)_4$-$C\boldsymbol{H_2}$-NH], 3.04 (m, 8H, 4×εCH_2*L-Lys*), 3.18-3.42 (br m, 18H, εCH_2*core*, 5×βCH_2*L-Trp*, O-CH_2-$C\boldsymbol{H_2}$-$NHSO_2$, NH-$C\boldsymbol{H_2}$-$C\boldsymbol{H_2}$-NH), 3.54, 3.65 (2m, 8H, 4×O-CH_2-$C\boldsymbol{H_2}$-NH), 3.81-3.94 [br m, 8H, O-$C\boldsymbol{H_2}$-$(CH_2)_4$-NH, O-$C\boldsymbol{H_2}$-CH_2-$NHSO_2$, 4×αCH *L-Lys*], 4.02-4.14 (br m, 12H, 4×O-$C\boldsymbol{H_2}$-CH_2-NH, 4×αCH*L-Trp*), 4.35 (m, 1H, αCH *core*), 4.59 (m, 1H, αCH *L-Trp*), 6.50 (m, 1H, C^4-H *Ph*), 6.67 (m, 2H, C^4-H *Ph*), 6.77 (m, 1H, $C^{2,6}$-H *Ph*), 6.92 (m, 2H, $C^{2,6}$-H *Ph*), 7.01 (m, 8H, $C^{2,6}$-H *Ph*, C^5-H *L-Trp*), 7.10 (m, 6H, $C^{2,6}$-H *L-Trp*), 7.21 (s, 4H, C^2-H *L-Trp*), 7.27 (d, *J* = 8.12 Hz, 1H, C^7-H *L-Trp*), 7.34-7.45 (br m, 8H, C^7-H *L-Trp*, $C^{3,5}$-H *SA*), 7.52 (d, *J* = 7.78 Hz, 1H, C^4-H *L-Trp*), 7.64 (m, 4H, C^4-H *L-Trp*), 7.88 (d, *J* = 7.95 Hz, 4H, $C^{2,6}$-H *SA*).

^{13}C NMR (500 MHz, MeOD):δ = 23.2, 24.2, 24.4 [γC, 2×O-$(CH_2)_2$-$\boldsymbol{C}H_2$-$(CH_2)_2$-NH], 28.3, 28.5, 28.9 (βC *L-Trp*), 29.5, 29.7, 30.0, 30.3 [δC,2×O-CH_2-$\boldsymbol{C}H_2$-CH_2-$\boldsymbol{C}H_2$-CH_2-NH], 32.0, 32.2 (βC), 37.8, 40.1, 40.2, 40.6, 40.8 (εC, 4×O-CH_2-$\boldsymbol{C}H_2$-NH, NH-$C\boldsymbol{H_2}$-$C\boldsymbol{H_2}$-NH), 43.5 (O-CH_2-$\boldsymbol{C}H_2$-$NHSO_2$), 43.9 [O-$(CH_2)_4$-$\boldsymbol{C}H_2$-NH], 54.4 (4×αC *L-Lys*), 55.2 (4×αC *L-Trp*), 56.1 (αC *L-Trp*), 56.4 (αC *core*), 67.7 (4×O-$\boldsymbol{C}H_2$-CH_2-NH), 68.1 (O-$\boldsymbol{C}H_2$-CH_2-$NHSO_2$), 69.2 [O-$\boldsymbol{C}H_2$-$(CH_2)_4$-NH], 105.8, 106.4 (C^4*Ph*), 107.0, 107.1, 107.3, 107.6 ($C^{2,6}$*Ph*), 108.1, 110.9 (C^3*L-Trp*), 112.5, 112.6 (C^7*L-Trp*), 119.4 (C^4*L-Trp*), 119.9, 120.2 (C^5*L-Trp*), 122.5 (C^6*L-Trp*), 122.9, 123.1 ($C^{3,5}$*SA*), 124.8, 125.7 (C^2*L-Trp*), 128.4, 128.6 (C^{3a}*L-Trp*), 130.0 ($C^{2,6}$*SA*), 136.8 (C^1*SA*), 137.3, 137.9 (C^1*Ph*), 138.1, 138.2 (C^{7a}*L-Trp*), 161.0, 161.2, 161.3, 161.6 ($C^{3,5}$*Ph*), 169.5(CONH *Ph*), 170.0, 170.5, 174.5 (CONH).

$[\alpha]_D^{25}$= +10.4 (c 1, MeOH).

M.p.: 196-204˚C.
